# The Role of Proteasome Beta Subunits in Gastrin-Mediated Transcription of *Plasminogen Activator Inhibitor-2* and *Regenerating Protein1*

**DOI:** 10.1371/journal.pone.0059913

**Published:** 2013-03-27

**Authors:** Adrian O’Hara, Alice Howarth, Andrea Varro, Rod Dimaline

**Affiliations:** Department of Cellular and Molecular Physiology, Institute of Translational Medicine, University of Liverpool, Liverpool, United Kingdom; University of California, Los Angeles, United States of America

## Abstract

The hormone gastrin physiologically regulates gastric acid secretion and also contributes to maintaining gastric epithelial architecture by regulating expression of genes such as *plasminogen activator inhibitor 2 (PAI-2)* and *regenerating protein 1(Reg1)*. Here we examine the role of proteasome subunit PSMB1 in the transcriptional regulation of *PAI-2* and *Reg1* by gastrin, and its subcellular distribution during gastrin stimulation. We used the gastric cancer cell line AGS, permanently transfected with the CCK2 receptor (AGS-GR) to study gastrin stimulated expression of *PAI-2* and *Reg1* reporter constructs when PSMB1 was knocked down by siRNA. Binding of PSMB1 to the *PAI-2* and *Reg1* promoters was assessed by chromatin immunoprecipitation (ChIP) assay. Subcellular distribution of PSMB1 was determined by immunocytochemistry and Western Blot. Gastrin robustly increased expression of *PAI-2* and *Reg1* in AGS-GR cells, but when PSMB1 was knocked down the responses were dramatically reduced. In ChIP assays, following immunoprecipitation of chromatin with a PSMB1 antibody there was a substantial enrichment of DNA from the gastrin responsive regions of the *PAI-2* and *Reg1* promoters compared with chromatin precipitated with control IgG. In AGS-GR cells stimulated with gastrin there was a significant increase in the ratio of nuclear:cytoplasmic PSMB1 over the same timescale as recruitment of PSMB1 to the *PAI-2* and *Reg1* promoters seen in ChIP assays. We conclude that PSMB1 is part of the transcriptional machinery required for gastrin stimulated expression of *PAI-2* and *Reg1*, and that its change in subcellular distribution in response to gastrin is consistent with this role.

## Introduction

The role of the hormone gastrin in the physiological regulation of gastric acid secretion is well established [1]. Additionally, there is increasing evidence to indicate that activation of the CCK2 receptor triggers a range of mechanisms that can be broadly categorized as associated with tissue defence and maintenance of gastric epithelial architecture [2]. These include epithelial cell proliferation [3], migration [4], invasion [5], tubulogenesis [6] and apoptosis [7]. Within the gastric epithelium, CCK2 receptors are primarily expressed by parietal cells and enterochromaffin-like (ECL) cells so that many of these responses are likely to result from paracrine cascades involving multiple cell types [2], [8]. Functional genomics approaches have identified a range of genes, whose expression is regulated by gastrin and that were hitherto unrecognized as targets of this hormone [9], [10], [11], [12].

There has been relatively good progress in elucidating the mechanisms by which gastrin physiologically regulates expression of genes involved in the acid secretory pathway such as *Histidine decarboxylase*, *Vesicular monoamine transporter2 (VMAT2)*and *chromogranin A*, which are key to ECL cell histamine synthesis and secretion [13], [14], [15], [16], [17], [18], [19]. Considerably less is known about the transcriptional mechanisms by which gastrin regulates genes that may be involved in the maintenance of gastric epithelial architecture. We recently identified a gastrin response element in the proximal promoter of the *VMAT2* gene, and showed that its activity was dependent on binding to a beta subunit of the 20S proteasome [15]. In the present study we sought to determine if other gastrin-regulated ECL cell genes, involved in maintenance of epithelial architecture were also dependent of proteasome subunits. We report here that the genes encoding regenerating protein 1 (Reg1) and plasminogen activator inhibitor type 2 (PAI-2) depend upon proteasome beta subunits for gastrin-mediated transcription. We also report that activation of the CCK2 receptor induces subcellular redistribution of proteasome beta subunit PSMB1, consistent with a transcriptional function.

## Materials and Methods

### Cells, Plasmids and Reagents

AGS cells stably transfected with full length cDNA for the human CCK2 receptor (AGS-GR) [18]were cultured in HAMS/F12 Nutrient mix media containing 10% FBS and 1% penicillin/streptomycin, and incubated at 37°C in a humidified atmosphere of 5.5% CO_2_/94.5% air.

A luciferase reporter construct containing 2340 bp of the human *PAI-2* promoter has been described previously [12]; a further construct containing 1.6 Kb of the PAI-2 promoter was generated using PCR on the 2340 bp template. Generation of 2111 bp luciferase reporter construct containing the rat *Reg1* promoter has been described previously [20]. Heptadecapeptide amidated gastrin (G17) was purchased from Bachem (St. Helens, UK); IL-8 and PD98059 were obtained from Calbiochem (Nottingham, UK); PGE2 was from Enzo Life Sciences (Exeter, UK), L740,093 was from Merck (West Drayton, UK) and 740-YP was from R&D systems (Abingdon, UK). All other chemicals were obtained from Sigma (Poole, UK).

### Immunocytochemistry

AGS-GR cells were cultured in a four-chamber culture slide (2×10^4^ cells per chamber) and incubated for 24 h. Following incubation, cells were treated with reagents as detailed in results, for up to 6 h. After treatment, the cells were fixed using paraformaldehyde (4%), permeabilized with Triton X-100 and processed for immunocytochemistry as previously described [21]. The proteasome subunits PSMB1 and PSMC1 were detected using primary rabbit polyclonal antibodies (Enzo) and PSMA5 was detected using a primary mouse monoclonal antibody (Enzo). All primary antibodies were used at a dilution of 1∶500, and visualized with either fluorescein-conjugated or Texas-Red-conjugated donkey anti-rabbit or anti-mouse secondary antibodies (Stratech, Soham, UK; 1∶400dilution), using an Axioplan 2 fluorescence microscope and AxioVision 4.6 software with deconvolution options (Carl Zeiss Microscopy, Cambridge, UK).

### Nuclear Extracts

Nuclear, cytoplasmic or total cell extracts were prepared from AGS-GR cells using a Nuclear Extraction Kit from Active Motif (La Hulpe, Belgium). Protein concentration was determined using a Bio-Rad DC protein assay (Bio-Rad, Hemel Hempstead, UK).

### Western Blots

Cytoplasmic (40 µg) and nuclear extracts (60–80 µg) were resolved using 10% SDS-PAGE gels, and the proteins transferred to Amersham Hybond ECL membrane (GE Healthcare Life Sciences, Little Chalfont, UK). Membranes were incubated with a primary rabbit polyclonal antibody for PSMB1 (Santa Cruz Biotechnology, Santa Cruz, USA) at a dilution of 1∶1000 overnight at 4°C. The following day the membrane was washed with TBS containing 0.1% Tween20, and incubated for one hour with a HRP-conjugated anti-rabbit secondary antibody (Santa Cruz), 1∶10000; the HRP was activated using the Immun-Star Western C Kit (Bio-Rad) and detection was carried out on a Bio-Rad ChemiDoc XRS+ with Image Lab V3.0 software (Bio-Rad). Membranes were re-blotted for PSMA5 and PSMC1 (Enzo), and finally HSP90 (cytoplasmic, Santa Cruz) or lamin (nuclear, Santa Cruz). Densitometry was performed using the Image Lab V3.0 software.

### Transfections, RNAi and Luciferase Assays

AGS-GR cells were cultured in six well plates (50,000 cells/well), 24 h post seeding the cells were transfected with validated siRNA for proteasome subunit or scrambled control (30 nM, Life Technologies, Paisley, UK) using the magnefect nano system (Nanotherics, Keele, UK) according to the manufacturer’s instructions. The settings used were; oscillation frequency, 2 Hz; displacement, 0.2 mm; 3600 cycles. Two days after siRNA transfection, the cells were transfected with the luciferase constructs for either *PAI-2* or *Reg1* (1 µg per well) and the constitutively active *renilla* reporter vector phRL-SV40 (Promega, Southampton, UK; 2.5 ng per well). After 24 h incubation, cells were treated with either serum free medium or 2 nM G17 in serum free medium for 6 h. Cells were then lysed and analysed using a dual luciferase assay system (Promega) according to the manufacturer’s instructions and luminescence determined using a Lumat LB9507 luminometer (Berthold, Redbourne, UK). Knockdown efficiency was assessed by western blot of whole cell extracts 72 h after transfection.

### ChIP Assays

ChIP assays were performed using the SimpleChIP Enzymatic Chromatin IP Kit (Magnetic beads), from Cell Signalling Technology (NEB, Hitchin, UK), according to the manufacturer’s instructions. Briefly, 4×10^7^ AGS-GR cells were seeded in four 15 cm dishes (1×10^7^ per dish) and incubated overnight. Cells were then treated with either 2×10^−9^ M G17 in serum free medium, or in serum free medium alone as a control, for two hours. Crosslinking of protein and DNA was achieved by adding formaldehyde to a final concentration of 1% for 10 min. DNA was digested using micrococcal nuclease (8000 gel units, 37°C, 20 min) and cell nuclei were lysed using a Bioruptor plus sonicator (Diagenode, Liege, Belgium; 7 pulses of 30 sec, with 30 sec rest at 4°C). Chromatin immunoprecipitation was carried out using an antibody for PSMB1 (Santa Cruz, sc-67345) and normal rabbit IgG to act as a negative control. DNA recovered from the immunoprecipitations was analysed by real time PCR on an Applied Biosystems 7500 system (Life Technologies) using a SYBR green mastermix with low Rox (Primer Design, Southampton, UK). Primers (Eurogentec, Southampton, UK) used for amplification were as follows: *PAI-2*, -198 (relative to the start of transcription), 5′-TCTTAAGTTTCAGAGTGACC-3′ and +51, 5′- TCTCTGAGTTGCTGTCTG-3′, *Reg1* -200, 5′-TGAGCAAGAGCAAAGTCCACCT and +18, 5′-CTGTAGGAGCTTTAATCAGGATCTGAGA-3′. The ΔΔCt algorithm was used to determine relative amplification in the PSMB1 immunoprecipitated samples compared to IgG, included in each PCR reaction.

## Results

We used the gastric cancer cell line AGS, transfected with the CCK2 (gastrin) receptor (AGS-GR, [18], which has been used extensively to characterize gastrin-stimulated gene transcription [2].

**Figure 1:**
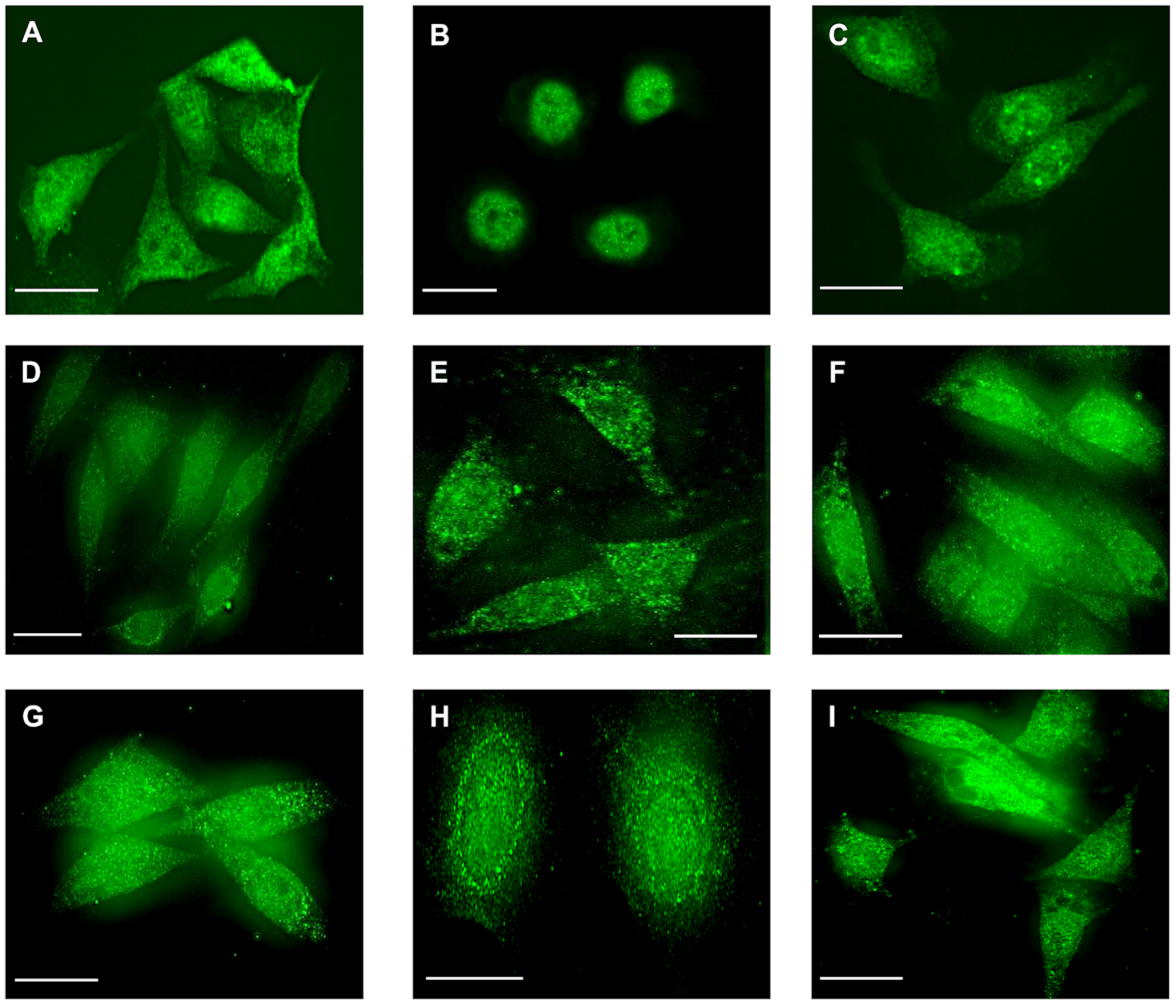
Localization of proteasome subunits in AGS-GR cells. A, PSMB1 in unstimulated cells; note cytoplasmic and nuclear location. B, PSMB1 in cells after 2 h exposure to gastrin (G17, 2×10^−9^ M); note absence of cytoplasmic staining. C, PSMB1 in cells 6 h after exposure to gastrin; note reappearance of cytoplasmic staining. D,E,F, localization of PSMA5 under conditions comparable to A,B,C, respectively; note retention of cytoplasmic localization during gastrin stimulation. G,H,I, localization of PSMC1 under conditions comparable to A,B,C, respectively; note retention of cytoplasmic localization during gastrin stimulation, with increased perinuclear staining. Scale bars, 20 µm.

**Figure 2:**
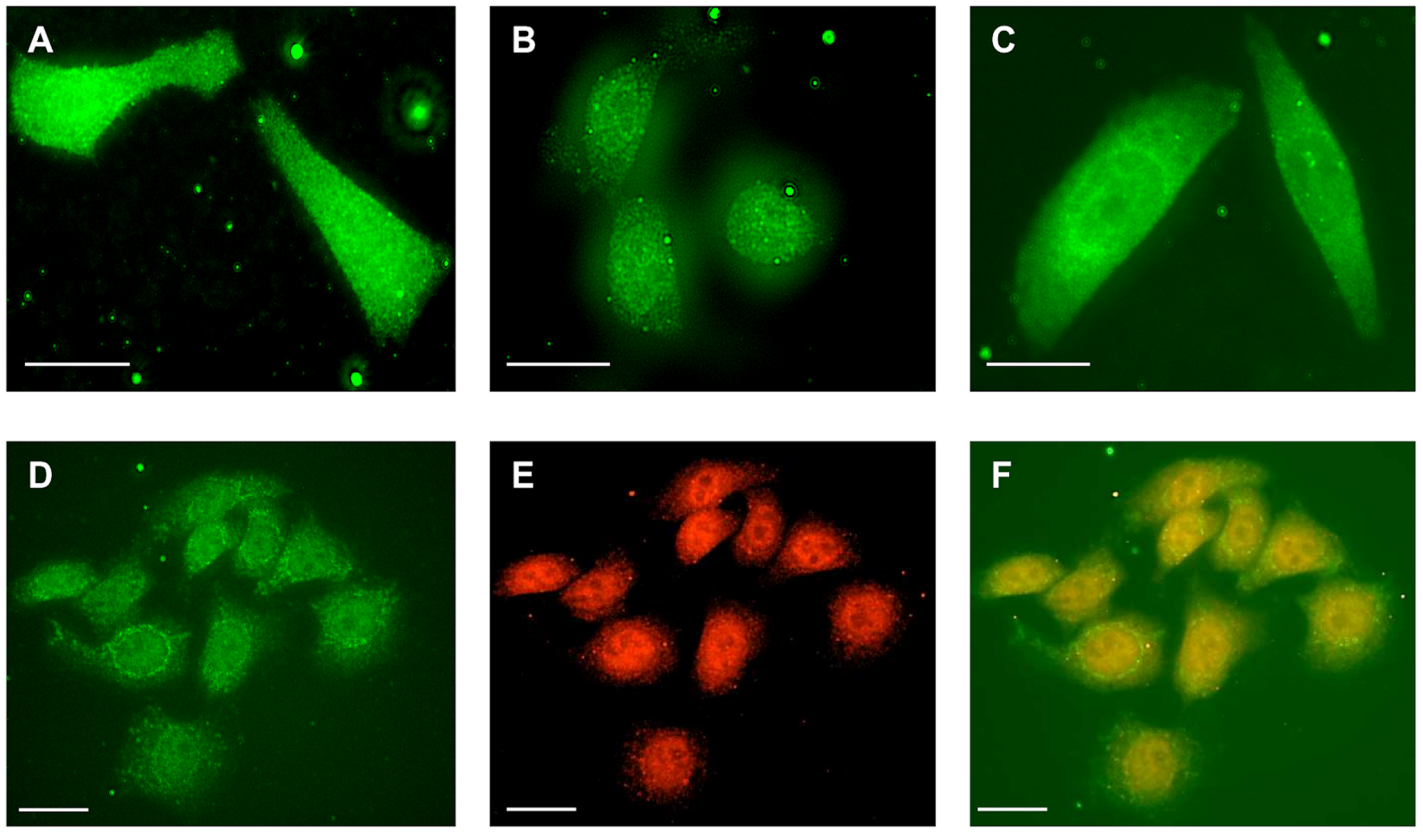
Localization of proteasome subunits in AGS-GR cells. **A**, PSMB1 in unstimulated AGS-GR cells; note cytoplasmic and nuclear localization. B, PSMB1 in AGS-GR cells following 2 h exposure to PMA (10^−7^ M); note reduction of cytoplasmic and intensification of nuclear staining. C, PSMB1 in AGS –GR cells following 2 h exposure to gastrin (G17, 2×10^−9^ M) in the presence of the PKC inhibitor Ro-32-0432 (10^−6^ M); note nuclear and cytoplasmic localization, comparable to unstimulated cells shown in A. D, PSMA5 (FITC) in AGS cells following 2 h exposure to gastrin (G17, 2×10^−9^ M); note retention of cytoplasmic staining with intensification in the perinuclear region. E, PSMB1(Texas red) in AGS cells following 2 h exposure to gastrin (G17, 2×10^−9^ M); note absence of cytoplasmic staining. F, overlay of D and E. Scale bars, 20 µm.

**Figure 3:**
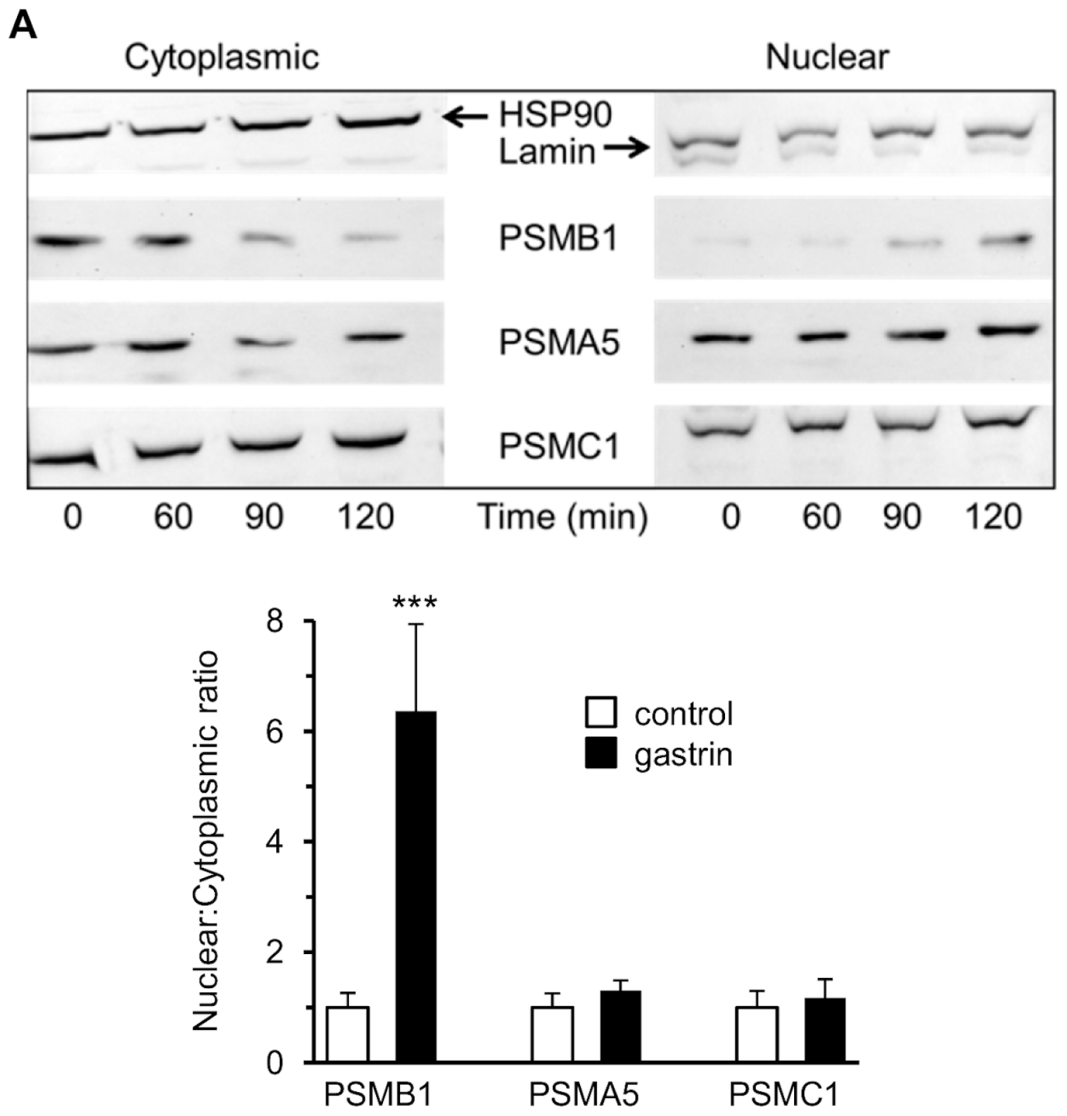
Nuclear and cytoplasmic abundance of proteasome subunits in gastrin-stimulated AGS-GR cells. A, representative western blot of proteasome subunits in cytoplasmic (left panel) and nuclear (right panel) extracts of AGS-GR cells, 0, 60, 90 or 120 minutes after stimulation with gastrin (G17, 2×10^−9^ M). Blots were re-probed with HSP90 (cytoplasmic) or lamin (nuclear). B, signals at 0 (control) and 120 (gastrin) minutes of gastrin stimulation were quantified by densitometry and normalized to HSP90 or lamin. Nuclear to cytoplasmic ratios at 0 min ( = 1.0) versus 120 min are shown for PSMB1, PSMA5 and PSMC1. ***, p<0.001; n = 7, ANOVA.

In unstimulated AGS-GR cells, immunoreactivity for proteasome beta subunit PSMB1 was observed throughout the cytoplasm and nucleus as previously described (Fig. 1) [15]. Similar distributions were also observed for an alpha subunit (PSMA5) and a regulatory subunit (PSMC1), (Fig. 1). After exposure to gastrin (G17, 2×10^−9^ M), within 1 h cytoplasmic staining of the beta subunit was reduced and after 2 h was virtually absent, while nuclear staining was retained or intensified (Fig. 1). After 6 h exposure to gastrin, subcellular distribution had reverted to that seen in control unstimulated cells (Fig. 1). In the case of the regulatory subunit PSMC1, and to a lesser extent the alpha subunit PSMA5, gastrin induced a partially perinuclear cytoplasmic localization, but there was no evidence of increased nuclear, or reduced cytoplasmic abundance (Figs. 1 and 2). Dual staining for PSMB1 and PSMA5 following gastrin stimulation clearly demonstrated disappearance of cytoplasmic PSMB1, whilst cytoplasmic PSMA5 was retained (Fig. 2). Gastrin had no effect in the presence of the CCK2 receptor antagonist L740093 (10^−7^ M). In order to further explore and quantify the subcellular distribution of proteasome subunits we prepared nuclear and cytoplasmic extracts of gastrin-stimulated or unstimulated AGS-GR cells and subjected them to Western blot for alpha, beta and regulatory proteasome subunits. In unstimulated cells, the nuclear: cytoplasmic ratio of PSMB1 (standardized to lamin and HSP90, respectively) was 1.67±0.43. After stimulation with gastrin (G17, 2×10^−9^ M for 2 h), the nuclear: cytoplasmic ratio was increased to 10.63±2.63 (p<0.001, ANOVA, n = 7). No changes were observed in nuclear: cytoplasmic ratios of PSMA5 (control 4.79±1.34, gastrin 6.14±0.98) or PSMC1 (control 3.76±1.13, gastrin 4.42±1.25) (Fig. 3).

In order to determine if activation of other GPCRs, or other receptor types might influence PSMB1 subcellular distribution we targeted a number of receptors reported to be functionally expressed in AGS cells. No effect on PSMB1 distribution was seen on activating the histamine H2 receptor [22] with 10^−5^ M histamine, the interleukin-8 receptor with 1.6×10^−8^ M IL-8 or the prostaglandin E2 receptor with 2.8×10^−5^ M PGE2 [23], [24]. PSMB1 was also unaffected by LPA at a dose (5×10^−5^ M) that stimulates branching morphogenesis in AGS-GR cells cultured on artificial basement membrane [6].

We also asked if functionally expressed tyrosine kinase receptors might activate PSMB1 redistribution. PSMB1 distribution was not altered by activation of the EGF receptor with EGF (8×10^−9^ M), a dose known to activate TFF1 expression in this cell type [25], and was unaffected by a second tyrosine kinase receptor, c-met, in response to 100 ng.ml^−1^ HGF.

Since previous studies indicate that many downstream effects of CCK2R activation are mediated by PKC [2] we used PMA as a receptor-independent activator of AGS-GR cells. When cells were stimulated with PMA (10^−7^ M) proteasome subunit PSMB1 underwent a subcellular redistribution similar to that seen in response to gastrin (Fig. 2). Moreover, the response to gastrin could be inhibited by application of the PKC inhibitor Ro-32-0432 (10^−6^ M) (Fig. 2). Gastrin may also act in part to activate PI3-kinase [26], but we found PSMB1 localization to be unaffected by the PI3-kinase activator, 740Y-P (2×10^−5^ M).

As well as its direct effects, gastrin induces paracrine activation of the *PAI-2* gene by multiple pathways involving the EGF and IL-8 receptors [23]. In the present study neither EGF nor PGE2 affected subcellular distribution of proteasome subunits, nevertheless we explored the possibility of paracrine activation perhaps involving different pathways. AGS-GR cells and AGS cells that constitutively express GFP (AGS-GFP) [27]were co-cultured in a 1∶1 ratio. When the co-culture was exposed to gastrin (G17, 10^−9^ M) for 2 h, AGS-GR cells demonstrated the previously described subcellular redistribution of PSMB1 whereas AGS-GFP cells were unaffected, suggesting that direct activation of the CCK2 receptor was required.

**Figure 4:**
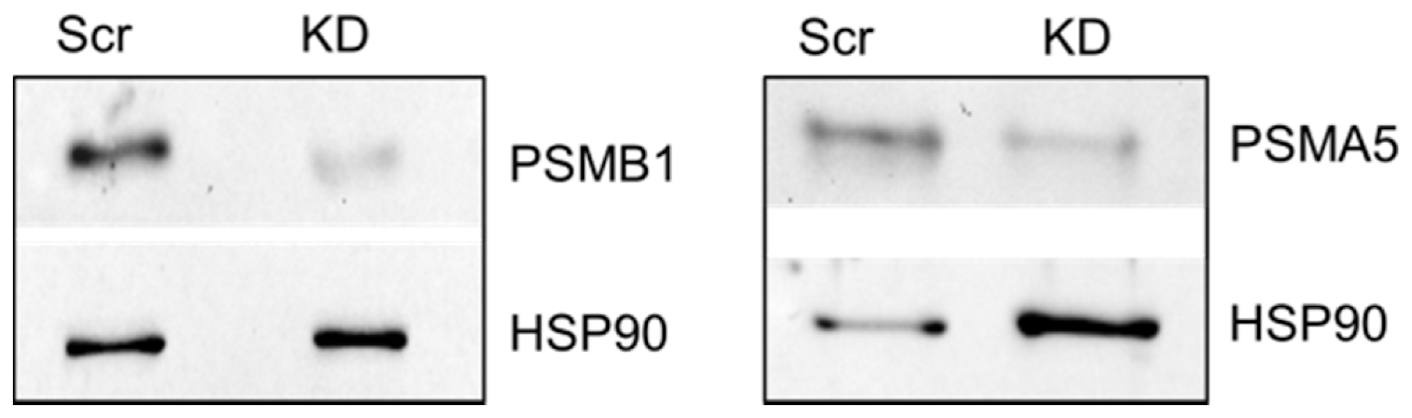
Knockdown of proteasome subunits in AGS-GR cells. Representative western blots of PSMB1 (left panel) and PSMA5 (right panel), in AGS-GR cells 72 hours after transfection with validated siRNA or scrambled control. Left lanes, scrambled control (Scr), right panels, knockdown (KD). Blots were re-probed for HSP90.

We have previously demonstrated gastrin responsiveness of *PAI-2* [12], [23] and *Reg1* [20], [28]. In order to determine the requirement for proteasome subunits in gastrin-stimulated transcription of these genes we knocked down PSMB1 or PSMA5 using RNA interference. Analysis by Western Blot indicated that 72 h following transfection of AGS-GR cells with PSMB1 siRNA, PSMB1 protein abundance was reduced to 31.2±5.5 (n = 3) per cent that seen in cells transfected with scrambled control RNA (Fig. 4). After transfection with PSMA5 siRNA, PSMA5 protein was reduced to 24.9±15.5 (n = 3) per cent that seen in cells transfected with scrambled RNA (Fig. 4).

**Figure 5:**
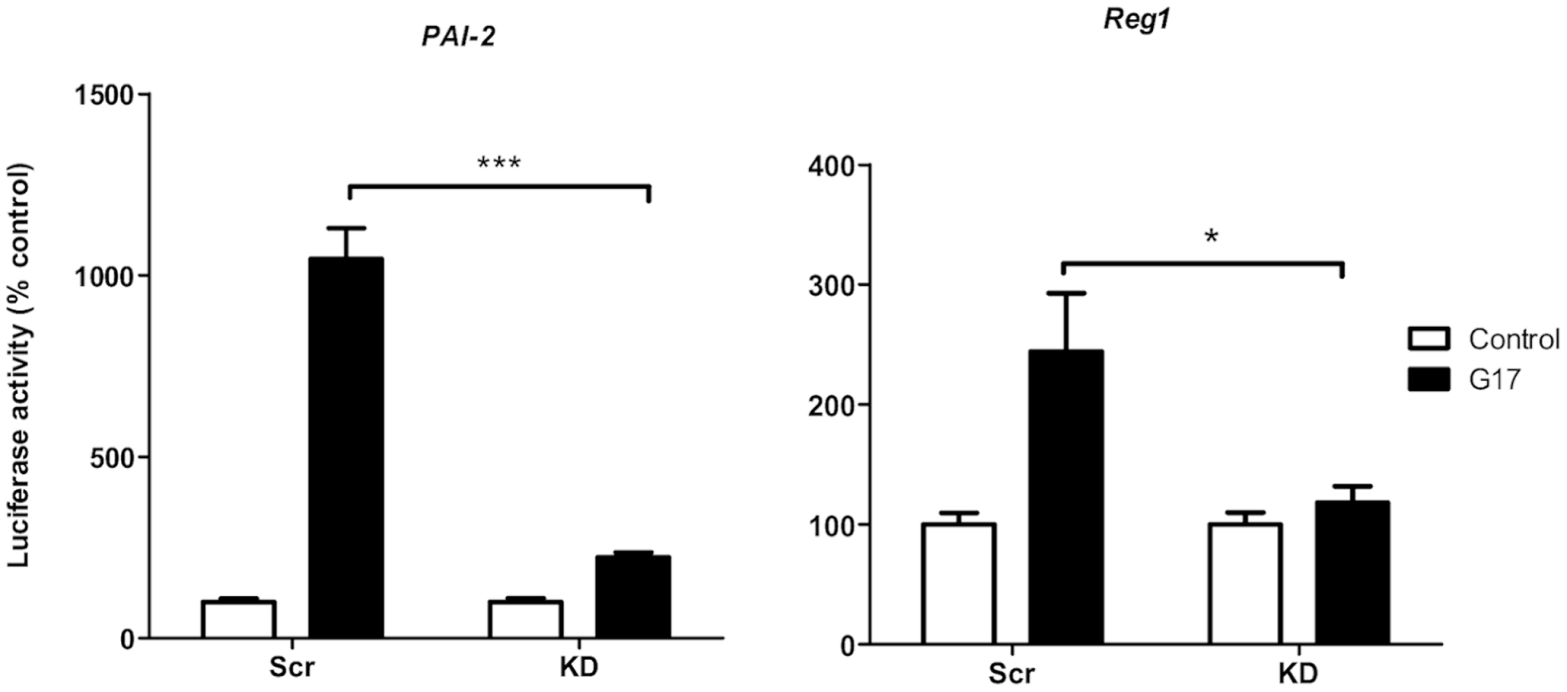
Gastrin-responsiveness of the *PAI-2* and *Reg1* promoters following PSMB1 knockdown. Response of the *PAI-2* (left panel) and *Reg1* (right panel) promoters to gastrin (G17, 2×10^−9^ M) in AGS cells transfected with PSMB1 siRNA (KD) or scrambled RNA (Scr). Open bars, unstimulated cells; closed bars, cells stimulated with gastrin (G17, 2×10^−9^ M) for 6 h. Values are mean ± SEM, n = 5–9. ***, p<0.001; *, p<0.05.

**Figure 6:**
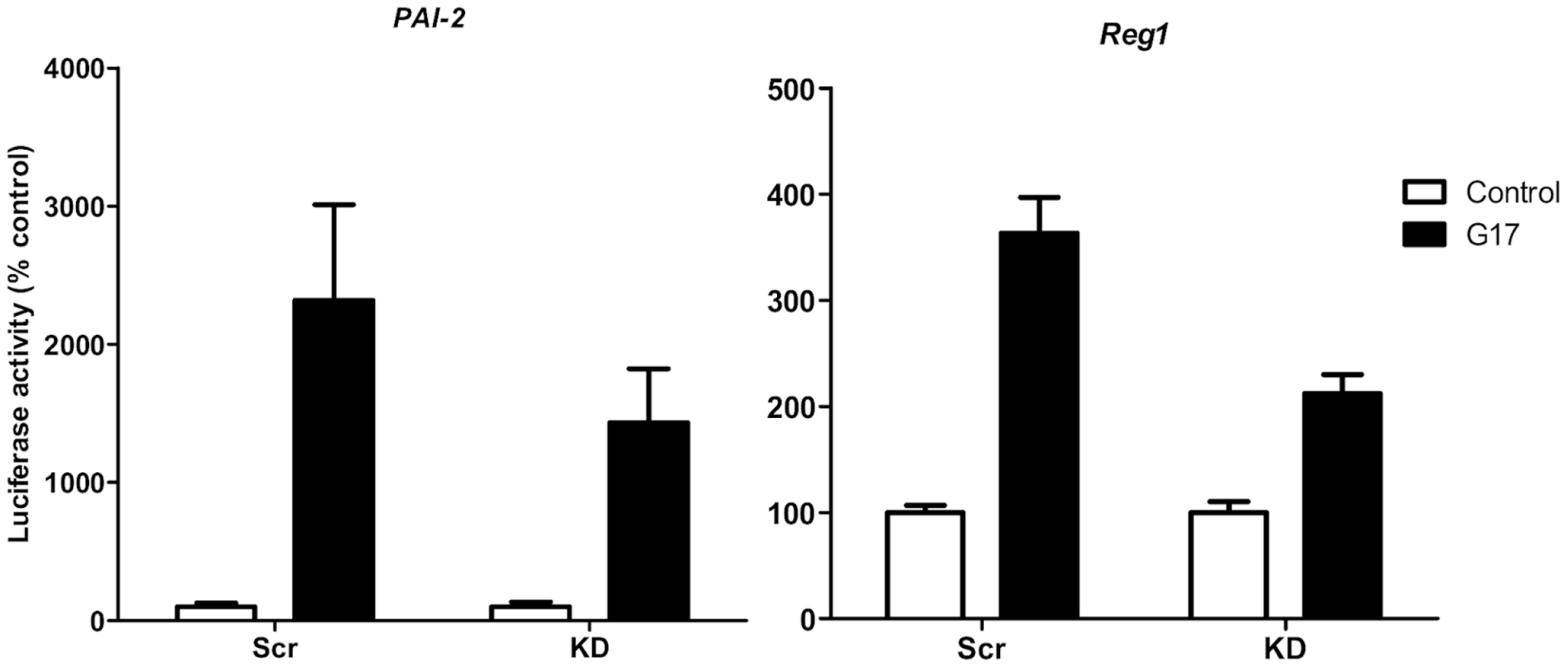
Gastrin-responsiveness of the *PAI-2* and *Reg1* promoters following PSMA5 knockdown. Response of the *PAI-2* (left panel) and *Reg1* (right panel) promoters to gastrin (G17, 2×10^−9^ M) in AGS cells transfected with PSMA5 siRNA (KD) or scrambled RNA (Scr). Open bars, unstimulated cells; closed bars, cells stimulated with gastrin (G17, 2×10^−9^ M) for 6 h. Values are mean ± SEM, n = 4–6.

In cells transfected with scrambled RNA and with a promoter-reporter construct containing 1.6 kb of the *PAI-2* promoter, treatment with gastrin (G17, 2×10^−9^ M) increased reporter activity 10.5±0.9 fold (n = 5) compared with unstimulated cells (Fig. 5). In contrast, in cells with PSMB1 knocked down, the response to gastrin of the *PAI-2* promoter was significantly reduced 5-fold (p<0.001, n = 5, ANOVA, Fig. 5). In cells with PSMA5 knocked down there was a modest reduction in the gastrin-responsiveness of *PAI-2* to 61.8±16.8 of that seen in cells transfected with scrambled RNA (Fig. 6).

In cells transfected with scrambled RNA and a promoter-reporter construct containing 2.1 kb of the *Reg1* promoter, treatment with gastrin increased reporter activity by 2.4±0.4 fold (n = 9) compared with unstimulated cells (Fig. 5). In contrast, in cells with PSMB1 knocked down, the response to gastrin of the *Reg1* promoter was virtually abolished (p<0.05, ANOVA, n = 9, Fig. 5). When PSMA5 was knocked down, there was a less marked reduction in the *Reg1* responsiveness to gastrin to 58.4% ±4.9% of that seen in cells transfected with scrambled RNA (Fig. 6).

In order to determine if there was binding of proteasome subunits to the *PAI-2* and *Reg1* promoters “*in vivo”* during gastrin stimulation we performed ChIP assays on AGS-GR cells using an anti PSMB1 antibody for immunoprecipitation, or IgG as a negative control. In three separate immunoprecipitation experiments, using chromatin from cells stimulated with gastrin (G17, 2×10^−9^ M for 2 h) we observed a 7.9±2.5 fold enrichment of DNA using primers that generated a 250 bp amplicon incorporating the region of the *PAI-2* promoter known to be responsive to gastrin [12], [23]. Using primers that generated a 218 bp amplicon including the gastrin responsive region of the *Reg1* promoter [20], [28], we observed a 10.0±4.6 fold enrichment. In two separate experiments using unstimulated cells there was no discernible enrichment of DNA (*PAI-2*, 0.17 and 0.3 fold; *Reg1*, 1.79 and 1.76 fold).

## Discussion

In the present study, we show that gastrin induces subcellular redistribution of the proteasome subunit PSMB1 from cytoplasm to nucleus. We also show that PSMB1 binds to the *PAI-2* and *Reg1* promoters *in vivo* and is required for gastrin-stimulated increases in expression of *PAI-2* and *Reg1*. These findings indicate that proteasome subunits form part of the transcriptional machinery to regulate expression of gastrin responsive ECL-cell genes that are involved in the maintenance of gastric mucosal integrity.

It is well established that the ubiquitin-proteasome pathway is important for modulating intracellular concentrations of a variety of generally short-lived molecules, including transcription factors [29], [30], [31], [32]. However, it is becoming increasingly clear that the proteasome is also able to regulate transcription by a variety of mechanisms not related to its proteolytic activity [33], [34], [35]. As part of these mechanisms, proteasome subunits may be recruited to regions of transcription, and this may involve elements of both the 20s catalytic core and the 19s regulatory complex [33], [36], [37], [38]. We previously reported that the proteasome subunit PSMB1, a component of the 20s central catalytic core of the proteasome, was recruited to the promoter of *VMAT2* in response to stimulation of AGS-GR cells by gastrin and was required for gastrin-stimulated *VMAT2* expression [15]. Binding of PSMB1 to a gastrin response element in the *VMAT2* promoter has subsequently been independently confirmed [39]. Moreover, PSMB1 has been shown to bind to the promoter and be required for activation of *retinol binding protein4* via a G4KA element, which taken with the present data indicates that this subunit can interact with a variety of DNA sequences within promoters to increase transcription. The nature of the physical interaction of PSMB1 and promoters remains to be established; conceivably it might act as a docking site for other proteins.

Proteasomes and their subunits are distributed throughout the cytoplasm and nucleus in most cell types [40] including the AGS-GR cells used in the present study [15]. One possibility is that PSMB1 already resident in the nucleus interacts with the *PAI-2* and *Reg1* promoters to upregulate transcription in response to activation of the CCK2 receptor. However, we found that CCK2R activation elicited a significant increase in the nuclear:cytoplasmic ratio of PSMB1 within the time frame that PSMB1 bound to the *PAI-*2 and *Reg1* promoters in ChIP studies. This raises the possibility that transcriptional regulation by PSMB1 in response to gastrin depends, at least in part, on movement of the subunit to the nucleus. Proteasomes appear to be imported into the nucleus as immature precursor complexes [41] and in some cases specific individual subunits may be imported preferentially [42]. Some alpha subunits contain recognizable nuclear localization signals [43], [44], but most subunits, including PSMB1, do not. It may be that PSMB1 has an as yet unrecognized NLS [39] or it may enter the nucleus combined with other proteins. Subcellular distribution of proteasomes varies with the cell cycle, particularly in association with alterations in the nuclear membrane [45], [46]. Nuclear localization of PSMB1 in response to GPCR activation has not been reported previously, although enhanced nuclear localization of proteasomes has been described in certain disease conditions [47], [48], [49] and in response to knockdown of GLUT4 [39]. Interestingly, knockdown of PSMA5 caused a modest reduction of gastrin-mediated *PAI-2* and *Reg1* transcription. Conceivably PSMA5 (or other subunits) may be involved with transport of PSMB1 to the nuclear compartment, without itself entering.

In many cases, signalling events downstream of the CCK2R are mediated by PKC [2], and in the present study, the gastrin-stimulated redistribution of PSMB1 was mimicked by PMA and prevented by the PKC inhibitor Ro-32-0432. CCK2R signalling may also occur via PI3-kinase [26], but in the present study an activator of this protein failed to affect PSMB1 localization. The specificity of the CCK2R response was demonstrated by the fact that it was prevented in the presence of a gastrin antagonist, and that a range of other GPCRs known to be functional on AGS-GR cells [6], [22], [23], [24] did not affect PSMB1 localization. PSMB1 was also unaffected by activation of the EGF receptor, which is consistent with the finding that PSMB1 is not required for activation of *TFF1* by either the EGF or CCK2 receptors in AGS-GR cells [15].

Functional genomics approaches have led to the recognition of hitherto unsuspected targets of gastrin, that are involved in maintaining function and integrity of the gastric epithelium [9], [10], [11], [12]. One such target gene, *PAI-2* is expressed in several cell types in the gastric mucosa including histamine-secreting ECL cells [12], [24]. It is an inhibitor of the urokinase plasminogen activator system [50], [51], and in the stomach is also increased by *Helicobacter pylori* and is associated with inhibition of cell invasion and suppression of apoptosis [12], [24].

Within the gastric epithelium, the *Reg1* gene is also a target of gastrin [20], [28], [52], [53] and is expressed in ECL cells as well as pepsinogen-secreting chief cells [53]. Gastric *Reg1* expression is increased by stress and by mucosal damage, and may act to alter the proportions of epithelial cell types [52], [54], [55]. The present data are therefore consistent with the idea that PSMB1 is part of the transcriptional machinery required for gastrin-stimulated expression of genes involved in the maintenance of gastric epithelial architecture. Interestingly the gastrin sensitive genes so far identified to be PSMB1-dependent, *PAI-2*, *Reg1*, and *VMAT2* [15] are all expressed in ECL cells, whilst TFF1 which did not demonstrate PSMB1 dependency [15], is not. It remains to be established if any gastrin sensitive genes expressed in cells other than ECL cells exhibit this mechanism.

## References

[1] Dockray GJ, Varro A, Dimaline R (1996) Gastric endocrine cells: gene expression, processing and targeting of active products. Physiological Reviews 76: 767–798. 8757788 10.1152/physrev.1996.76.3.767

[2] Dockray G, Dimaline R, Varro A (2005) Gastrin: old hormone, new functions. Pflügers Archiv European Journal of Physiology 449: 344–355. 15480747 10.1007/s00424-004-1347-5

[3] Wang TC, Koh TJ, Varro A, Cahill RJ, Dangler CA, et al (1996) Processing and proliferative effects of human progastrin in transgenic mice. J Clin Invest 98: 1918–1929. 8878444 10.1172/JCI118993 PMC507632

[4] Noble PJ, Wilde G, White MR, Pennington SR, Dockray GJ, et al (2003) Stimulation of gastrin-CCKB receptor promotes migration of gastric AGS cells via multiple paracrine pathways. Am J Physiol 284: G75–G84. 10.1152/ajpgi.00300.2002 12488236

[5] Wroblewski LE, Pritchard DM, Carter S, Varro A (2002) Gastrin-stimulated gastric epithelial cell invasion: the role and mechanism of increased matrix metalloproteinase 9 expression. Biochem J 365: 873–879. 11971760 10.1042/BJ20020068 PMC1222716

[6] Pagliocca A, Wroblewski LE, Ashcroft FJ, Noble PJ, Dockray GJ, et al (2002) Stimulation of the gastrin-cholecystokinin(B) receptor promotes branching morphogenesis in gastric AGS cells. Am J Physiol 283: G292–G299. 10.1152/ajpgi.00056.2002 12121875

[7] Todisco A, Ramamoorthy S, Witham T, Pausawasdi N, Srinivasan S, et al (2001) Molecular mechanisms for the antiapoptotic action of gastrin. Am J Physiol Gastrointest Liver Physiol 280: G298–307. 11208554 10.1152/ajpgi.2001.280.2.G298

[8] Dimaline R, Varro A (2007) Attack and defence in the gastric epithelium - a delicate balance. Exp Physiol 92: 591–601. 17412751 10.1113/expphysiol.2006.036483

[9] Khan ZE, Wang TC, Cui G, Chi AL, Dimaline R (2003) Transcriptional regulation of the human trefoil factor, TFF1, by gastrin1. Gastroenterology 125: 510–521. 12891554 10.1016/s0016-5085(03)00908-9

[10] Fjeldbo CS, Bakke I, Erlandsen SE, Holmseth J, Laegreid A, et al (2012) Gastrin upregulates the prosurvival factor secretory clusterin in adenocarcinoma cells and in oxyntic mucosa of hypergastrinemic rats. Am J Physiol Gastrointest Liver Physiol 302: G21–33. 21995960 10.1152/ajpgi.00197.2011

[11] Pagliocca A, Hegyi P, Venglovecz V, Rackstraw SA, Khan Z, et al (2008) Identification of ezrin as a target of gastrin in immature mouse gastric parietal cells. Exp Physiol 93: 1174–1189. 18567601 10.1113/expphysiol.2008.042648

[12] Varro A, Hemers E, Archer D, Pagliocca A, Haigh C, et al (2002) Identification of plasminogen activator inhibitor-2 as a gastrin- regulated gene: Role of Rho GTPase and menin. Gastroenterology 123: 271–280. 12105855 10.1053/gast.2002.34162

[13] Dimaline R, Evans D, Forster ER, Sandvik AK, Dockray GJ (1993) Control of gastric corpus chromogranin A messenger RNA abundance in the rat. Am J Physiol 264: G583–588. 8460709 10.1152/ajpgi.1993.264.3.G583

[14] Dimaline R, Sandvik AK, Evans D, Forster ER, Dockray GJ (1993) Food stimulation of histidine decarboxylase messenger RNA abundance in rat gastric fundus. J Physiol 465: 449–458. 8229845 10.1113/jphysiol.1993.sp019686 PMC1175439

[15] Catlow K, Ashurst HL, Varro A, Dimaline R (2007) Identification of a gastrin response element in the vesicular monoamine transporter type 2 promoter and requirement of 20 S proteasome subunits for transcriptional activity. J Biol Chem 282: 17069–17077. 17442673 10.1074/jbc.M611421200

[16] Raychowdhury R, Schafer G, Fleming J, Rosewicz S, Wiedenmann B, et al (2002) Interaction of early growth response protein 1 (Egr-1), specificity protein 1 (Sp1), and cyclic adenosine 3′5′-monophosphate response element binding protein (CREB) at a proximal response element is critical for gastrin-dependent activation of the chromogranin A promoter. Mol Endocrinol 16: 2802–2818. 12456801 10.1210/me.2001-0292

[17] Dimaline R, Struthers J (1996) Expression and regulation of a vesicular monoamine transporter in rat stomach: a putative histamine transporter. J Physiol 490 (Pt 1): 249–256. 10.1113/jphysiol.1996.sp021140 PMC1158661 8745292

[18] Watson F, Kiernan RS, Deavall DG, Varro A, Dimaline R (2001) Transcriptional activation of the rat vesicular monoamine transporter 2 promoter in gastric epithelial cells: regulation by gastrin. Journal of Biol Chem 276: 7661–7671. 11113118 10.1074/jbc.M006697200

[19] Raychowdhury R, Fleming JV, McLaughlin JT, Bulitta CJ, Wang TC (2002) Identification and characterization of a third gastrin response element (GAS-RE3) in the human histidine decarboxylase gene promoter. Biochem Biophys Res Commun 297: 1089–1095. 12372397 10.1016/s0006-291x(02)02345-8

[20] Ashcroft FJ, Varro A, Dimaline R, Dockray GJ (2004) Control of expression of the lectin-like protein Reg-1 by gastrin: role of the Rho family GTPase RhoA and a C-rich promoter element. Biochem J 381: 397–403. 15109306 10.1042/BJ20031793 PMC1133845

[21] Blackmore CG, Varro A, Dimaline R, Bishop L, Gallacher DV, et al (2001) Measurement of secretory vesicle pH reveals intravesicular alkalinization by vesicular monoamine transporter type 2 resulting in inhibition of prohormone cleavage. J Physiol 531: 605–617. 11251044 10.1111/j.1469-7793.2001.0605h.x PMC2278512

[22] Ancha HR, Kurella RR, Stewart CA, Damera G, Ceresa BP, et al (2007) Histamine stimulation of MMP-1(collagenase-1) secretion and gene expression in gastric epithelial cells: role of EGFR transactivation and the MAP kinase pathway. Int J Biochem Cell Biol 39: 2143–2152. 17656145 10.1016/j.biocel.2007.06.003

[23] Almeida-Vega S, Catlow K, Kenny S, Dimaline R, Varro A (2009) Gastrin activates paracrine networks leading to induction of PAI-2 via MAZ and ASC-1. Am J Physiol Gastrointest Liver Physiol 296: G414–423. 19074642 10.1152/ajpgi.90340.2008 PMC2643906

[24] Varro A, Noble PJ, Pritchard DM, Kennedy S, Hart CA, et al (2004) Helicobacter pylori induces plasminogen activator inhibitor 2 (PAI-2) in gastric epithelial cells through NF-kB and RhoA: implications for invasion and apoptosis Cancer Res. 64: 1695–1702. 10.1158/0008-5472.can-03-2399 14996729

[25] Khan ZE, Wang TC, Cui G, Chi AL, Dimaline R (2003) Transcriptional regulation of the human trefoil factor, TFF1, by gastrin. Gastroenterology 125: 510–521. 12891554 10.1016/s0016-5085(03)00908-9

[26] Daulhac L, Kowalski-Chauvel A, Pradayrol L, Vaysse N, Seva C (1999) Src-family tyrosine kinases in activation of ERK-1 and p85/p110- phosphatidylinositol 3-kinase by G/CCKB receptors. J Biol Chem 274: 20657–20663. 10400698 10.1074/jbc.274.29.20657

[27] Varro A, Noble PJ, Wroblewski LE, Bishop L, Dockray GJ (2002) Gastrin-cholecystokinin(B) receptor expression in AGS cells is associated with direct inhibition and indirect stimulation of cell proliferation via paracrine activation of the epidermal growth factor receptor. Gut 50: 827–833. 12010885 10.1136/gut.50.6.827 PMC1773243

[28] Steele IA, Dimaline R, Pritchard DM, Peek RM Jr, Wang TC, et al (2007) Helicobacter and gastrin stimulate Reg1 expression in gastric epithelial cells through distinct promoter elements. Am J Physiol Gastrointest Liver Physiol 293: G347–354. 17463184 10.1152/ajpgi.00076.2007

[29] Akiyama H, Kamitani T, Yang X, Kandyil R, Bridgewater LC, et al (2005) The transcription factor Sox9 is degraded by the ubiquitin-proteasome system and stabilized by a mutation in a ubiquitin-target site. Matrix Biology 23: 499–505. 15694126 10.1016/j.matbio.2004.10.002

[30] Ciechanover A, DiGiuseppe JA, Bercovich B, Orian A, Richter JD, et al (1991) Degradation of nuclear oncoproteins by the ubiquitin system in vitro. Proc Natl Acad Sci USA 88: 139–143. 1846034 10.1073/pnas.88.1.139 PMC50765

[31] Lingbeck JM, Trausch-Azar JS, Ciechanover A, Schwartz AL (2005) E12 and E47 modulate cellular localization and proteasome-mediated degradation of MyoD and Id1. Oncogene 24: 6376–6384. 16007194 10.1038/sj.onc.1208789

[32] Nakagawa K, Yokosawa H (2000) Degradation of transcription factor IRF-1 by the ubiquitin-proteasome pathway: The C-terminal region governs the protein stability. FEBS Journal 267: 1680–1686. 10.1046/j.1432-1327.2000.01163.x 10712599

[33] Geng F, Wenzel S, Tansey WP (2012) Ubiquitin and proteasomes in transcription. Annu Rev Biochem 81: 177–201. 22404630 10.1146/annurev-biochem-052110-120012 PMC3637986

[34] Lipford JR, Deshaies RJ (2003) Diverse roles for ubiquitin-dependent proteolysis in transcriptional activation. Nat Cell Biol 5: 845–850. 14523392 10.1038/ncb1003-845

[35] Muratani M, Tansey WP (2003) How the ubiquitin-proteasome system controls transcription. Nature Reviews Molecular Cell Biology 4: 192–201. 12612638 10.1038/nrm1049

[36] Auld KL, Brown CR, Casolari JM, Komili S, Silver PA (2006) Genomic association of the proteasome demonstrates overlapping gene regulatory activity with transcription factor substrates. MolCell 21: 861–871. 10.1016/j.molcel.2006.02.020 16543154

[37] Gonzalez F, Delahodde A, Kodadek T, Johnston SA (2002) Recruitment of a 19S proteasome subcomplex to an activated promoter. Science 296: 548–550. 11964484 10.1126/science.1069490

[38] Sikder D, Johnston SA, Kodadek T (2006) Widespread, but non-identical, association of proteasomal 19 and 20 S proteins with yeast chromatin. J Biol Chem 281: 27346–27355. 16837462 10.1074/jbc.M604706200

[39] Inoue E, Yamashita A, Inoue H, Sekiguchi M, Shiratori A, et al (2010) Identification of glucose transporter 4 knockdown-dependent transcriptional activation element on the retinol binding protein 4 gene promoter and requirement of the 20 S proteasome subunit for transcriptional activity. J Biol Chem 285: 25545–25553. 20530491 10.1074/jbc.M109.079152 PMC2919119

[40] Enenkel C, Lehmann A, Kloetzel P-M (1998) Subcellular distribution of proteasomes implicates a major location of protein degradation in the nuclear envelope-ER network in yeast. EMBO J 17: 6144–6154. 9799224 10.1093/emboj/17.21.6144 PMC1170941

[41] von Mikecz A (2006) The nuclear ubiquitin-proteasome system. J Cell Sci 119: 1977–1984. 16687735 10.1242/jcs.03008

[42] Klare N, Seeger M, Janek K, Jungblut PR, Dahlmann B (2007) Intermediate-type 20 S Proteasomes in HeLa Cells: “Asymmetric” Subunit Composition, Diversity and Adaptation. J Mol Biol 373: 1–10. 17804016 10.1016/j.jmb.2007.07.038

[43] Tanaka K, Yoshimura T, Tamura T, Fujiwara T, Kumatori A, et al (1990) Possible mechanism of nuclear translocation of proteasomes. FEBS Lett 271: 41–46. 2226812 10.1016/0014-5793(90)80367-r

[44] Nederlof PM, Wang HR, Baumeister W (1995) Nuclear localization signals of human and Thermoplasma proteasomal alpha subunits are functional in vitro. Proc Natl Acad Sci USA 92: 12060–12064. 8618844 10.1073/pnas.92.26.12060 PMC40296

[45] Reits EA, Benham AM, Plougastel B, Neefjes J, Trowsdale J (1997) Dynamics of proteasome distribution in living cells. EMBO J 16: 6087–6094. 9321388 10.1093/emboj/16.20.6087 PMC1326292

[46] Palmer A, Mason GG, Paramio JM, Knecht E, Rivett AJ (1994) Changes in proteasome localization during the cell cycle. Eur J Cell Biol 64: 163–175. 7957305

[47] Nakamura A, Kitami T, Mori H, Mizuno Y, Hattori N (2006) Nuclear localization of the 20S proteasome subunit in Parkinson’s disease. Neurosci Lett 406: 43–48. 16911859 10.1016/j.neulet.2006.07.050

[48] Kim H-D, Tomida A, Ogiso Y, Tsuruo T (1999) Glucose-regulated stresses cause degradation of DNA topoisomerase IIα by inducing nuclear proteasome during G1 cell cycle arrest in cancer cells. J Cell Physiol 180: 97–104. 10362022 10.1002/(SICI)1097-4652(199907)180:1<97::AID-JCP11>3.0.CO;2-Y

[49] Ogiso Y, Tomida A, Tsuruo T (2002) Nuclear Localization of Proteasomes Participates in Stress-inducible Resistance of Solid Tumor Cells to Topoisomerase II-directed Drugs. Cancer Research 62: 5008–5012. 12208754

[50] Kruithof EK, Tran-Thang C, Gudinchet A, Hauert J, Nicoloso G, et al (1987) Fibrinolysis in pregnancy: a study of plasminogen activator inhibitors. Blood 69: 460–466. 2432970

[51] Wohlwend A, Belin D, Vassalli JD (1987) Plasminogen activator-specific inhibitors produced by human monocytes/macrophages. The J Exptl Med 165: 320–339. 2434595 10.1084/jem.165.2.320 PMC2188521

[52] Fukui H, Kinoshita Y, Maekawa T, Okada A, Waki S, et al (1998) Regenerating gene protein may mediate gastric mucosal proliferation induced by hypergastrinemia in rats. Gastroenterology 115: 1483–1493. 9834276 10.1016/s0016-5085(98)70027-7

[53] Higham AD, Bishop LA, Dimaline R, Blackmore CG, Dobbins AC, et al (1999) Mutations of RegIalpha are associated with enterochromaffin-like cell tumor development in patients with hypergastrinemia. Gastroenterology 116: 1310–1318. 10348814 10.1016/s0016-5085(99)70495-6

[54] Asahara M, Mushiake S, Shimada S, Fukui H, Kinoshita Y, et al (1996) Reg gene expression is increased in rat gastric enterochromaffin-like cells following water immersion stress. Gastroenterology 111: 45–55. 8698224 10.1053/gast.1996.v111.pm8698224

[55] Kazumori H, Ishihara S, Hoshino E, Kawashima K, Moriyama N, et al (2000) Neutrophil chemoattractant 2 beta regulates expression of the Reg gene in injured gastric mucosa in rats. Gastroenterology 119: 1610–1622. 11113082 10.1053/gast.2000.20262

